# Microstructure and Mechanical Properties of Hot-Rolled ZrAl_14_Ti_3_ and ZrAl_14_Ti_9_ Alloys

**DOI:** 10.3390/ma18194459

**Published:** 2025-09-24

**Authors:** Xing Zhang, Yujing Yang, Mingchao Yang, Wang Li, Zhixin Li

**Affiliations:** College of Physical Science and Technology, Hebei Normal University of Science & Technology, Qinhuangdao 066004, China

**Keywords:** zirconium alloys, microstructure, mechanical properties, high strength

## Abstract

This study systematically investigated the microstructure and mechanical properties of hot-rolled and quenched ZrAl_14_Ti_3_ and ZrAl_14_Ti_9_ (at.%) alloys. Microstructural analysis revealed that both alloys consisted of equiaxed α-Zr and Zr_3_Al grains. Increasing Ti content lowered the dissolution temperature of Zr_3_Al in α-Zr, enhancing the solubility of Al in α-Zr under identical thermal conditions and decreasing the Zr_3_Al phase fraction. Moreover, higher Ti content in the ZrAl_14_Ti_9_ alloy significantly promoted Zr_3_Al recrystallization and α-Zr globularization, leading to grain refinement and complete elimination of the α-Zr basal texture. Mechanical property evaluation showed that the ZrAl_14_Ti_3_ alloy exhibited offset yield and tensile strengths of 888 ± 12 MPa and 1056 ± 19 MPa, respectively, with a fracture elongation of 23 ± 1%. The ZrAl_14_Ti_9_ alloy displayed enhanced strength without compromising ductility, achieving a 110 MPa increase in offset yield strength (998 ± 6 MPa) while maintaining the same fracture elongation (23 ± 2%). The strengthening effects observed in the ZrAl_14_Ti_9_ alloy stemmed from multiple synergistic mechanisms: solid-solution strengthening due to increased Ti content in α-Zr, refinement of both Zr_3_Al and α-Zr grains, a higher proportion of the harder α-Zr phase, and orientation hardening resulting from the elimination of the α-Zr basal texture.

## 1. Introduction

Zirconium (Zr) alloys are widely used as structural materials in nuclear reactors due to their exceptional corrosion resistance, low thermal neutron absorption cross-section, and excellent high-temperature mechanical properties [1]. Additionally, their low density and thermal expansion coefficient render them promising candidates for aerospace applications [2]. However, currently commercialized Zr alloys, primarily designed for nuclear applications with strict constraints on thermal neutron economy and corrosion resistance, are limited in the variety and concentration of alloying elements, with the hexagonal close-packed (HCP) α-Zr dominating their microstructure [1,2,3,4,5]. The α-Zr phase exhibits a high critical resolved shear stress (CRSS) for activating <a + c>-type slip and twinning at room temperature, which accommodate strain along the c-axis [6]. This intrinsic characteristic results in insufficient slip and twinning system activation during deformation, failing to meet the von Mises criterion (requiring five independent slip systems for homogeneous deformation), and consequently leads to inferior plasticity compared to high-symmetry phases such as face-centered cubic (FCC) and body-centered cubic (BCC) structures. Therefore, achieving a balanced strength–ductility synergy in α-Zr alloys remains challenging. Coarse-grained pure Zr exhibits a tensile elongation of up to 24%, but its tensile strength is limited to approximately 380 MPa [7]. Solid-solution strengthening, grain refinement, and interface strengthening can increase the strength of α-Zr alloys to 800 MPa; however, this is typically accompanied by a significant reduction in ductility (<10%) [7]. For non-nuclear applications, where alloying constraints are less stringent than in the nuclear industry, simultaneous enhancement of strength and ductility may be achieved by introducing ductile secondary phases into α-Zr matrix. Inspired by titanium (Ti) alloy design principles, incorporating BCC-structured β-Zr phase into α-Zr alloys was initially considered a viable strategy. Although Zr and Ti belong to the same group with identical outer electron configurations, their alloying behaviors differ significantly [8]. While β-stabilizing elements can effectively retain the metastable β-phase in Ti alloys through quenching, their introduction in Zr alloys typically induces the formation of intermetallic compounds or the ω-phase, even under rapid cooling rates (>100 °C/s) [8]. This fundamental discrepancy suggests that β-Zr may not serve as an ideal ductile phase in Zr-based alloys.

The Zr_3_Al phase, possessing an L1_2_ structure, differs from other intermetallics by exhibiting an increase in electrical resistivity with rising temperature, indicative of a metallic nature [9]. Schulson conducted systematic studies on Zr_3_Al-based alloys, revealing that, in addition to good corrosion resistance and low neutron absorption, Zr_3_Al deforms at room temperature via *a*/3<211> partial dislocations, accompanied by stacking faults, demonstrating reasonable plastic deformability, albeit with limited strength [10]. Therefore, the Zr_3_Al phase could potentially serve as a ductile phase in α-Zr alloys to enhance plasticity. Moreover, aluminum (Al), as an economical industrial metal, not only significantly strengthens α-Zr but also reduces alloy density and raw material costs [11]. Chen et al. demonstrated that adding 14 at.% Al to a ZrSn_1_._5_ (at.%) alloy introduced a substantial Zr_3_Al phase fraction, achieving simultaneous enhancement of strength and ductility [12].

Ti exhibits complete solid solubility in Zr at room temperature, providing effective solid-solution strengthening while reducing material density and cost [8]. Based on this background, the present study investigated the microstructure and mechanical properties of hot-rolled ZrAl_14_Ti_3_ (at.%) and ZrAl_14_Ti_9_ (at.%) alloys, focusing on the effects of Ti content variation on their strengthening mechanisms.

## 2. Experimental Procedure

Both alloys were fabricated from industrial-grade raw materials, including commercially pure Zr (Zr + Hf ≥ 99.8 wt.%), Al (99.9 wt.%), and Ti (99.9 wt.%). Each alloy was synthesized by non-consumable arc melting under an argon atmosphere, followed by six remelting cycles to ensure chemical homogeneity. The as-cast ingots were homogenized at 800 °C for 30 min in a muffle furnace, followed by multi-pass rolling with 5 min interpass holding times. A total thickness reduction of 60% was achieved prior to water quenching. Phase identification of the hot-rolled and water-quenched alloys was performed using X-ray diffraction (XRD, Rigaku SmartLab, Tokyo, Japan). Crystallographic orientation mapping was conducted using a Zeiss GeminiSEM 450 field-emission scanning electron microscope (FEG-SEM, Oberkochen, Germany) equipped with an EDAX Velocity electron backscatter diffraction (EBSD, Mahwah, NJ, USA) detector. Identical acquisition parameters were employed for comparative analysis: an accelerating voltage of 20 kV, a step size of 0.5 μm, and a scan area of 60 μm × 50 μm. The EBSD datasets for both alloys were reindexed using the spherical indexing algorithm, with over 97% of the points in each dataset exhibiting confidence index (CI) values above 0.1. All crystallographic analyses were performed using OIM Analysis™ 9.1 software. The EBSD specimens were prepared from the transverse direction (TD plane) of the hot-rolled alloys by mechanical polishing, followed by electrochemical polishing in a solution of 10 vol% perchloric acid in methanol at 20 V and −20 °C for 120 s. Electron channeling contrast imaging (ECCI) was carried out using a Hitachi SU7000 FEG-SEM (Hino, Japan). The ECCI specimen (TD plane) was prepared by mechanical polishing. Quasi-static tensile tests at room temperature were conducted following the ASTM E8/E8M standard [13] guidelines using an Instron 5982 universal testing machine (Norwood, MA, USA) with dog-bone specimens having gauge dimensions of 20 × 4 × 2 mm^3^. The tests were performed at an initial strain rate of 0.5 × 10^−3^ s^−1^, with strain measured using a mechanical extensometer with a 10 mm gauge length.

## 3. Results and Discussion

### 3.1. XRD Results

**Figure 1:**
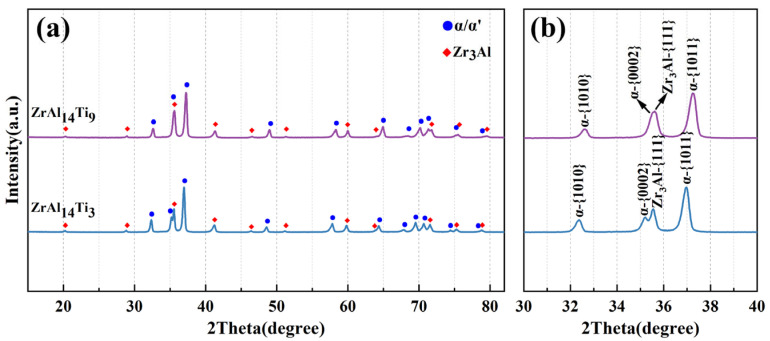
(**a**) XRD patterns and (**b**) magnified view (2θ = 30–40°) for hot-rolled ZrAl_14_Ti_3_ and ZrAl_14_Ti_9_ alloys.

Figure 1a displays the XRD patterns of the hot-rolled ZrAl_14_Ti_3_ and ZrAl_14_Ti_9_ alloys, while Figure 1b presents a magnified view of the 2θ range from 30° to 40°. Phase analysis confirmed the presence of α-Zr and Zr_3_Al phases in both alloys, with no detectable diffraction peaks corresponding to the β-phase (BCC) or other metastable phases. Furthermore, a distinct shift in the α-Zr diffraction peaks toward higher angles was observed in the magnified patterns with increasing Ti content. This peak shift is attributed to changes in the lattice parameters of the solid-solution phase caused by the incorporation of solute atoms. The atomic radii of Ti and Al are smaller than that of Zr. Their incorporation into the α-Zr matrix reduces the lattice parameters, resulting in the observed peak shift. The extent of the peak shift correlates with the solute content: a greater displacement toward higher angles indicates higher concentrations of Ti or Al atoms dissolved in the α-Zr lattice.

### 3.2. Microstructure Evolution

**Figure 2:**
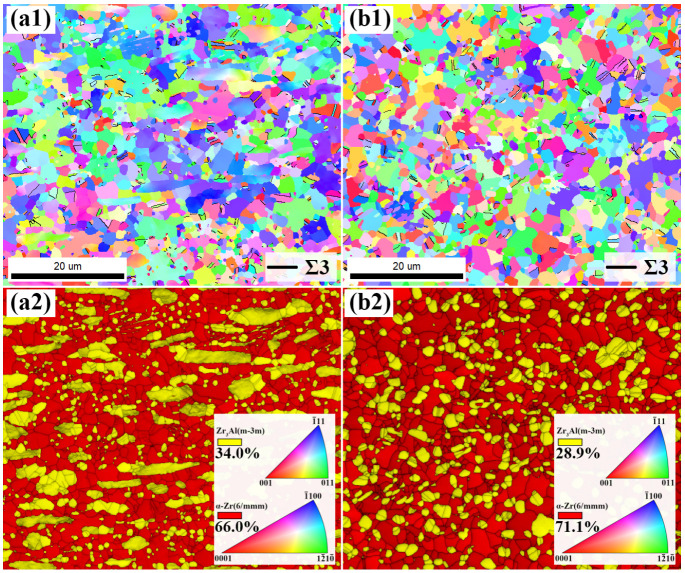
(**a1**,**a2**) Inverse pole figure map and confidence index-overlaid phase map of hot-rolled ZrAl_14_Ti_3_ alloy, and (**b1**,**b2**) corresponding maps of ZrAl_14_Ti_9_ alloy.

Figure 2(a1) shows the inverse pole figure (IPF) map of the hot-rolled ZrAl_14_Ti_3_ alloy, and Figure 2(a2) presents the corresponding phase map overlaid with CI values. The alloy exhibits a dual-phase microstructure consisting of equiaxed α-Zr and Zr_3_Al grains, with phase fractions of 66.0 vol.% α-Zr and 34.0 vol.% Zr_3_Al. A high density of 60°/<111> twin boundaries was observed within the Zr_3_Al phase. According to Gagné, these twin boundaries originate from stacking faults introduced during recrystallization, confirming that the Zr_3_Al phase underwent dynamic recrystallization during hot rolling [14]. Moreover, the Zr_3_Al grains exhibit two distinct morphologies: (i) elongated coarse grains (>10 μm in length) and (ii) fine equiaxed grains ranging from sub-micron size to a few micrometers. Figure 2(b1,b2) present the IPF map and the CI-overlaid phase map of the hot-rolled ZrAl_14_Ti_9_ (at.%) alloy, respectively. Similarly to the ZrAl_14_Ti_3_ alloy, the ZrAl_14_Ti_9_ alloy also exhibits an equiaxed α-Zr + Zr_3_Al dual-phase microstructure, but with a lower Zr_3_Al fraction of 28.9 vol.%. The increase in Ti content suppresses the precipitation temperature of Zr_3_Al from α-Zr, thereby increasing the solid solubility of Al in the α-Zr matrix at the rolling temperature and reducing the equilibrium Zr_3_Al fraction. In the ZrAl_14_Ti_9_ alloy, the Zr_3_Al grains are more uniformly distributed, with fewer coarse elongated grains compared to those in the ZrAl_14_Ti_3_ alloy. In addition, 60°/<111> twin boundaries remain prevalent in the Zr_3_Al phase. Quantitative analysis shows that the Zr_3_Al phase in the ZrAl_14_Ti_3_ alloy has a twin boundary density of 0.509 μm^−1^ (518.80 μm boundary length/1020 μm^2^ area), whereas the ZrAl_14_Ti_9_ alloy exhibits a higher value of 0.609 μm^−1^ (528.3 μm/867 μm^2^). The increased twin boundary density indicates a higher degree of recrystallization in the Zr_3_Al phase of the ZrAl_14_Ti_9_ alloy.

**Figure 3:**
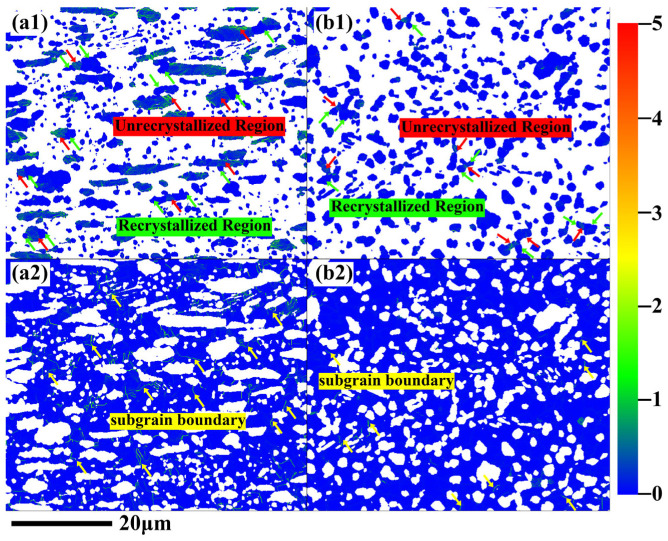
(**a1**,**a2**) Kernel average misorientation (KAM) maps of Zr_3_Al and α-Zr phases in hot-rolled ZrAl_14_Ti_3_ alloy, and (**b1**,**b2**) corresponding KAM maps in ZrAl_14_Ti_9_ alloy.

**Figure 4:**
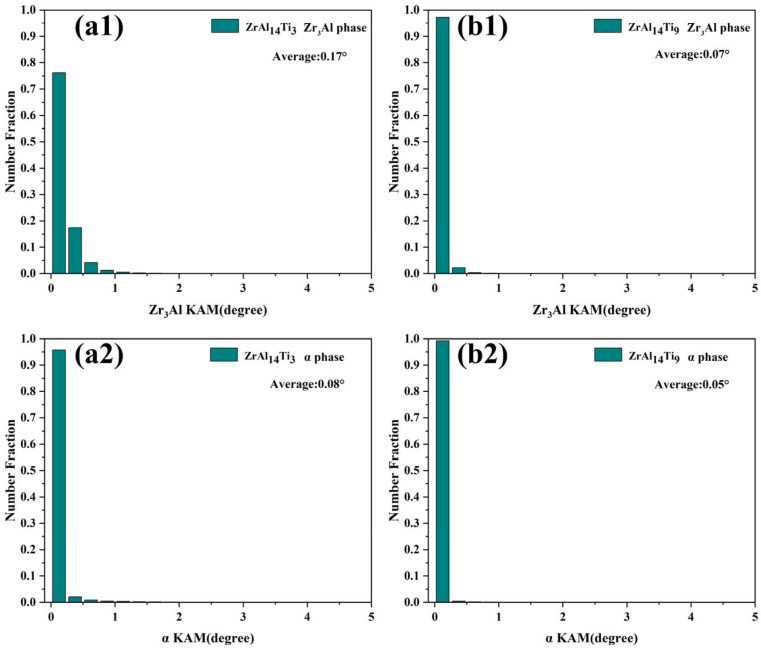
Statistical distribution charts of kernel average misorientation (KAM) values: (**a1**) Zr_3_Al phase in ZrAl_14_Ti_3_ alloy, (**b1**) Zr_3_Al phase in ZrAl_14_Ti_9_ alloy, (**a2**) α-Zr phase in ZrAl_14_Ti_3_ alloy, (**b2**) α-Zr phase in ZrAl_14_Ti_9_ alloy.

Figure 3 presents the kernel average misorientation (KAM) maps of the Zr_3_Al and α-Zr phases in the ZrAl_14_Ti_3_ and ZrAl_14_Ti_9_ alloys. In the ZrAl_14_Ti_3_ alloy, coarse and elongated Zr_3_Al grains (Figure 3(a1)) exhibit higher KAM values with an inhomogeneous distribution; regions of significantly elevated KAM (indicated by red arrows) alternate with areas of relatively low KAM (marked by green arrows). Fine and equiaxed Zr_3_Al grains consistently exhibit lower KAM values. The Zr_3_Al phase in the ZrAl_14_Ti_9_ alloy shows similar KAM distribution characteristics (Figure 3(b1)): coarse grains retain high and unevenly distributed KAM values, whereas fine grains exhibit uniformly low values. These results indicate that dynamic recrystallization has extensively occurred in the fine Zr_3_Al grains of both alloys, whereas coarse grains have undergone partial or no recrystallization. In the ZrAl_14_Ti_3_ alloy (Figure 3(a2)), the α-Zr contains a high density of subgrain boundaries (highlighted by yellow arrows), whereas the ZrAl_14_Ti_9_ alloy (Figure 3(b2)) exhibits significantly fewer such boundaries. Globularization of α-Zr in Ti and Zr alloys is a strain-induced geometric transformation fundamentally different from the conventional nucleation–growth mechanism of dynamic recrystallization. It proceeds through three stages: (i) kinking and shear-band formation within α-lamellae, (ii) subdivision of lamellae by low-angle dislocation walls, and (iii) globularization driven by minimization of interfacial energy [15,16,17,18,19]. During the second stage, low-angle boundaries (LABs) form as geometrically necessary dislocations accumulate into walls that subdivide the lamellae. High-angle grain boundaries (HABs) develop through progressive misorientation accumulation within these walls. Therefore, it is concluded that the α-Zr in the ZrAl_14_Ti_9_ alloy has experienced a more advanced stage of globularization compared with that in the ZrAl_14_Ti_3_ alloy. Quantitative KAM analysis (Figure 4) shows a marked decrease in the average KAM value of the Zr_3_Al phase, from 0.17° in the ZrAl_14_Ti_3_ alloy to 0.07° in the ZrAl_14_Ti_9_ alloy. In contrast, the average KAM values of the α-Zr decrease only slightly, from 0.08° to 0.05°. Although subgrain boundaries exhibit high KAM values, the low KAM within α-Zr grains in the ZrAl_14_Ti_3_ alloy results in only a marginal increase in the overall average KAM.

**Figure 5:**
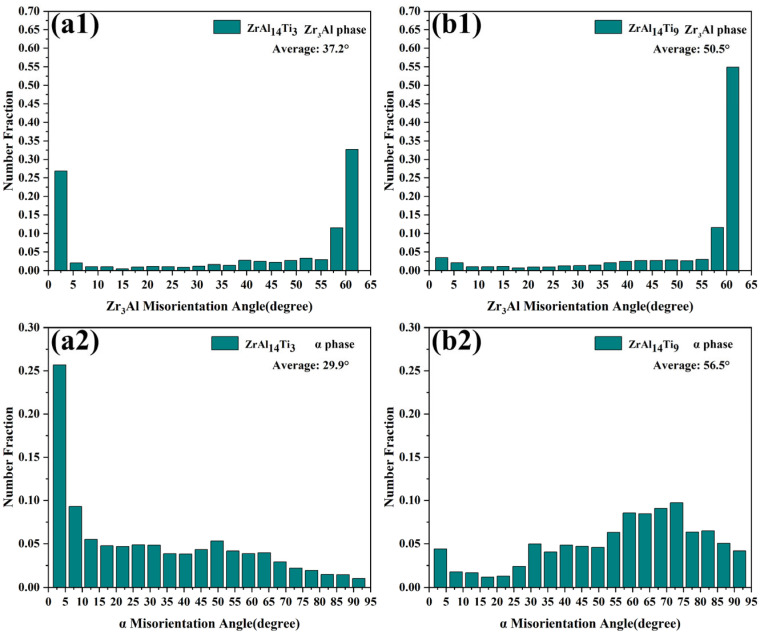
Misorientation angle distribution charts: (**a1**) Zr_3_Al phase in ZrAl_14_Ti_3_ alloy, (**b1**) Zr_3_Al phase in ZrAl_14_Ti_9_ alloy, (**a2**) α-Zr phase in ZrAl_14_Ti_3_ alloy, (**b2**) α-Zr phase in ZrAl_14_Ti_9_ alloy.

**Figure 6:**
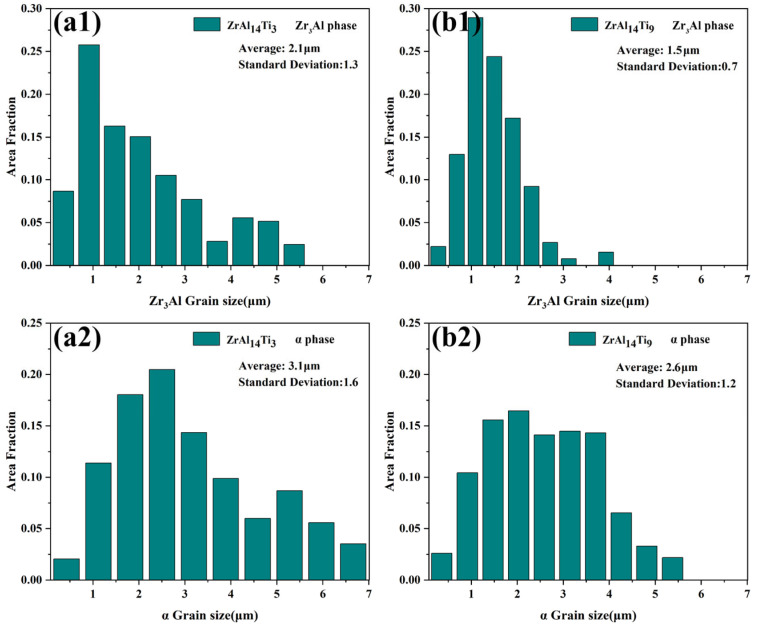
Grain size statistical charts: (**a1**) Zr_3_Al phase in ZrAl_14_Ti_3_ alloy, (**b1**) Zr_3_Al phase in ZrAl_14_Ti_9_ alloy, (**a2**) α-Zr phase in ZrAl_14_Ti_3_ alloy, (**b2**) α-Zr phase in ZrAl_14_Ti_9_ alloy.

Figure 5(a1,b1) presents the misorientation angle distributions of the Zr_3_Al phase in the ZrAl_14_Ti_3_ and ZrAl_14_Ti_9_ alloys, respectively. Both alloys exhibit a pronounced peak near 60°, corresponding to the high density of 60°/<111> twin boundaries. The ZrAl_14_Ti_9_ alloy exhibits a markedly greater fraction of 60° misorientations, whereas the ZrAl_14_Ti_3_ alloy contains a higher proportion of subgrain boundaries (<5°). This indicates that the Zr_3_Al phase in the ZrAl_14_Ti_3_ alloy underwent a lower degree of recrystallization, preserving numerous subgrain boundaries generated during thermomechanical processing. For the α-Zr phase (Figure 5(a2,b2)), the ZrAl_14_Ti_3_ alloy exhibits a higher fraction of low-angle subgrain boundaries, whereas the ZrAl_14_Ti_9_ alloy is dominated by high-angle grain boundaries. This finding is consistent with the KAM results. Figure 6 presents the grain size statistics for the α-Zr and Zr_3_Al phases in both alloys. In the ZrAl_14_Ti_3_ alloy, the α-Zr exhibits an average grain size of 3.1 ± 1.6 µm, while the Zr_3_Al phase has an average grain size of 2.1 ± 1.3 µm. In the ZrAl_14_Ti_9_ alloy, the corresponding grain sizes are 2.6 ± 1.2 µm for α-Zr and 1.5 ± 0.7 µm for Zr_3_Al. The enhanced extent of recrystallization and globularization contributes to this grain refinement.

**Figure 7:**
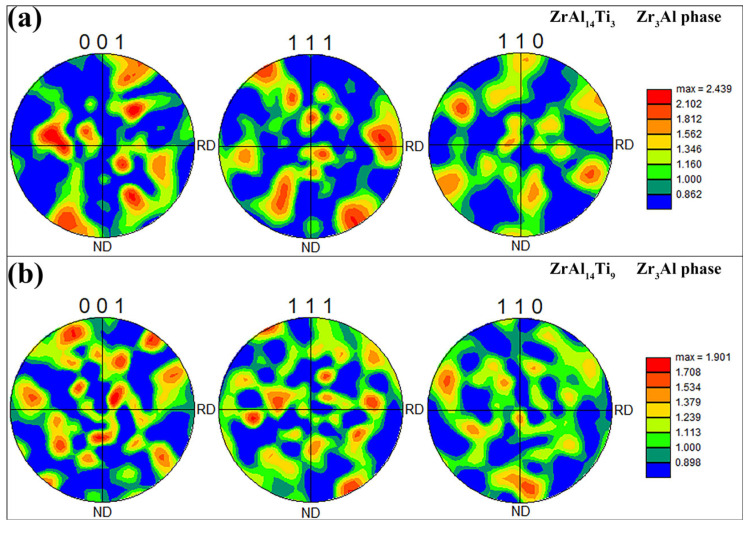
{001}, {111} and {110} pole figures of Zr_3_Al phase: (**a**) ZrAl_14_Ti_3_ alloy, (**b**) ZrAl_14_Ti_9_ alloy.

**Figure 8:**
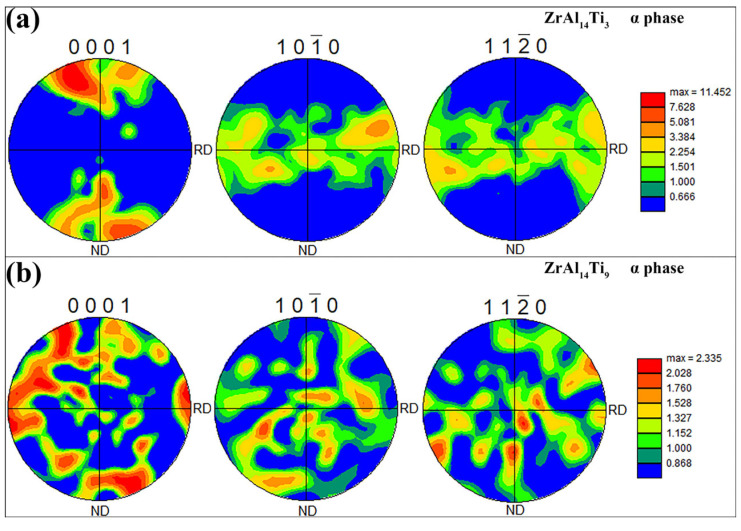
{0001}, {10$\bar{1}$0} and {11$\bar{2}$0} pole figures of α-Zr phase: (**a**) ZrAl_14_Ti_3_ alloy, (**b**) ZrAl_14_Ti_9_ alloy.

**Figure 9:**
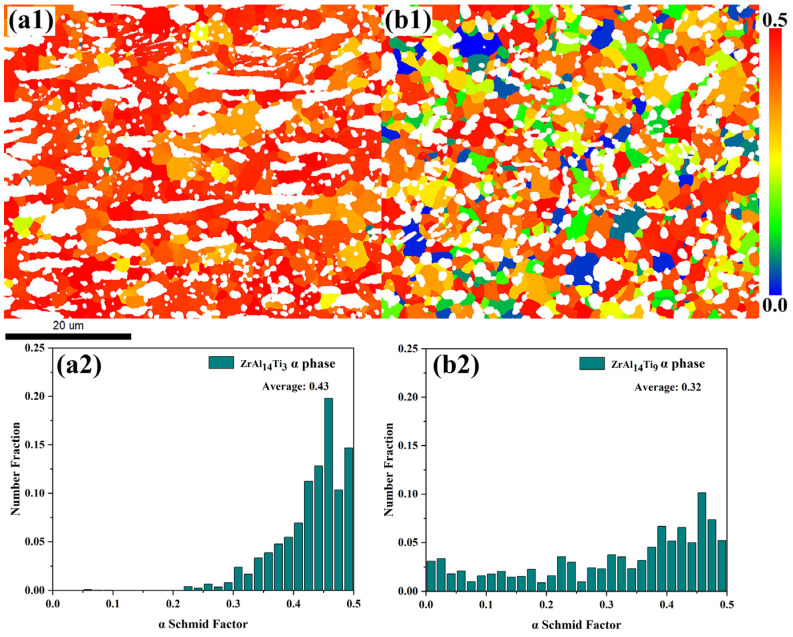
Schmid factor analysis for prismatic slip in α-Zr phase: (**a1**) Map of ZrAl_14_Ti_3_ alloy, (**a2**) Statistical distribution chart of ZrAl_14_Ti_3_ alloy, (**b1**) Map of ZrAl_14_Ti_9_ alloy, (**b2**) Statistical distribution chart of ZrAl_14_Ti_9_ alloy.

Figure 7a,b presents the pole figures of the Zr_3_Al phase in the ZrAl_14_Ti_3_ and ZrAl_14_Ti_9_ alloys, respectively. In both alloys, the Zr_3_Al exhibits a uniform orientation distribution, indicating the absence of pronounced texture. Figure 8a,b presents the pole figures of the α-Zr phase in the ZrAl_14_Ti_3_ and ZrAl_14_Ti_9_ alloys. In the ZrAl_14_Ti_3_ alloy, the α-Zr exhibits a strong basal texture, with a peak multiple of random distribution (MRD) value of 11.45. In contrast, the ZrAl_14_Ti_9_ alloy exhibits a much weaker texture, with a peak MRD of only 2.34, indicating no significant crystallographic preference. The basal texture in α-Zr alloys during hot rolling develops through an initial stage dominated by {1 0 −1 2} tensile twinning, which reorients the c-axis towards the normal direction (ND) and generates high-stored-energy twin boundaries that serve as preferential nucleation sites, followed by recrystallization-driven selective growth of ND-oriented grains, resulting in texture sharpening [19,20]. In the ZrAl_14_Ti_9_ alloy, subgrain boundaries within the α-Zr evolved into high-angle boundaries through dislocation absorption, resulting in the elimination of the basal texture. Figure 9(a1,b1) presents the Schmid factor maps for prismatic slip of the α-Zr phase in the ZrAl_14_Ti_3_ and ZrAl_14_Ti_9_ alloys, respectively, while Figure 9(a2,b2) shows the corresponding statistical distributions. The average Schmid factor for the ZrAl_14_Ti_3_ alloy is 0.43, significantly higher than the 0.32 measured for the ZrAl_14_Ti_9_ alloy. The lower Schmid factor of the ZrAl_14_Ti_9_ alloy indicates orientation hardening, attributed to the elimination of the basal texture.

**Figure 10:**
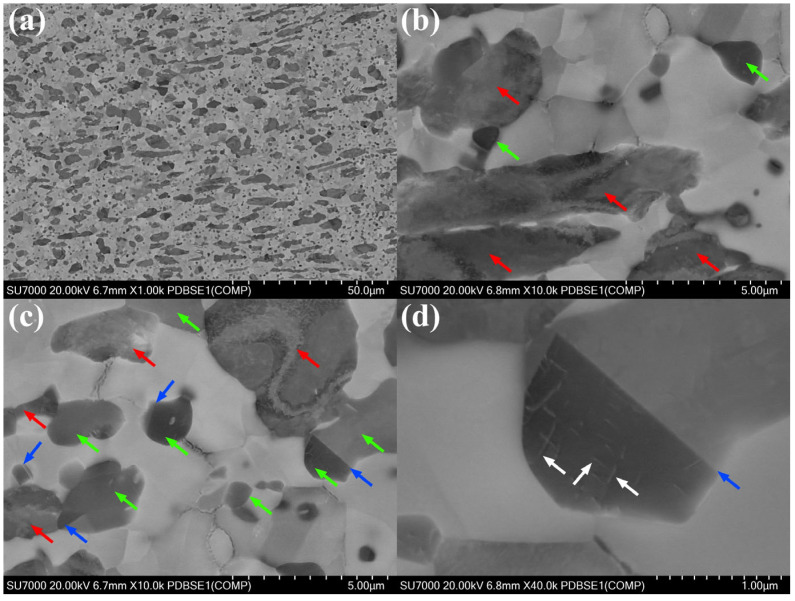
ECCI images of ZrAl_14_Ti_3_ alloy: (**a**) 1000× magnification, (**b**) 10,000× magnification, (**c**) 10,000× magnification, (**d**) 40,000× magnification.

**Figure 11:**
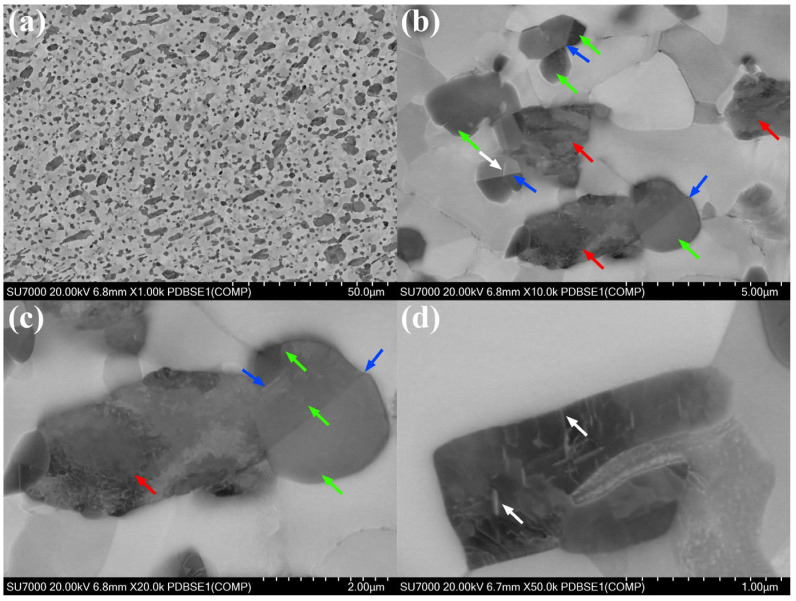
ECCI images of ZrAl_14_Ti_9_ alloy: (**a**) 1000× magnification, (**b**) 10,000× magnification, (**c**) 20,000× magnification, (**d**) 50,000× magnification.

Figure 10 and Figure 11 show the ECCI images of the ZrAl_14_Ti_3_ and ZrAl_14_Ti_9_ alloys at different magnifications. The low-magnification images reveal that both alloys contain coarse elongated and fine equiaxed Zr_3_Al grains. Higher-magnification images show that the α-Zr grains in both alloys exhibit a low dislocation density. Within the coarse Zr_3_Al grains of both alloys, some regions remain unrecrystallized (indicated by red arrows), containing a high density of dislocations, whereas other regions are recrystallized (indicated by green arrows) and contain fewer dislocations. These recrystallized and unrecrystallized regions within the coarse Zr_3_Al grains correspond, respectively, to the low- and high-KAM regions observed in the KAM maps. Furthermore, stacking faults (indicated by white arrows) and twin boundaries (indicated by blue arrows) are observed within the recrystallized Zr_3_Al grains in both alloys.

The influence of Ti content on the microstructure of ZrAl_14_-based alloys is primarily attributed to its effect on the initial martensitic transformation behavior. According to previous studies on Zr-Ti martensitic transformations [21,22], increasing Ti content induces a significant transition in the as-quenched microstructure. At lower Ti concentrations, the structure consists predominantly of dislocated lath martensite characterized by coarse packets of similarly oriented laths separated by low-angle boundaries. In contrast, higher Ti content promotes the formation of twinned plate martensite comprising fine, acicular plates belonging to multiple crystallographic variants separated by high-angle boundaries. This fundamental difference in initial microstructure profoundly affects subsequent thermomechanical processing. The fine, crystallographically diverse plate structure provides numerous high-energy nucleation sites for recrystallization, facilitating more complete recrystallization while inhibiting the development of strong texture and coarse macrozones. Consequently, alloys with higher Ti content exhibit enhanced recrystallization kinetics, finer equiaxed grains, and more randomized texture compared to their low-Ti counterparts.

### 3.3. Mechanical Properties

**Figure 12:**
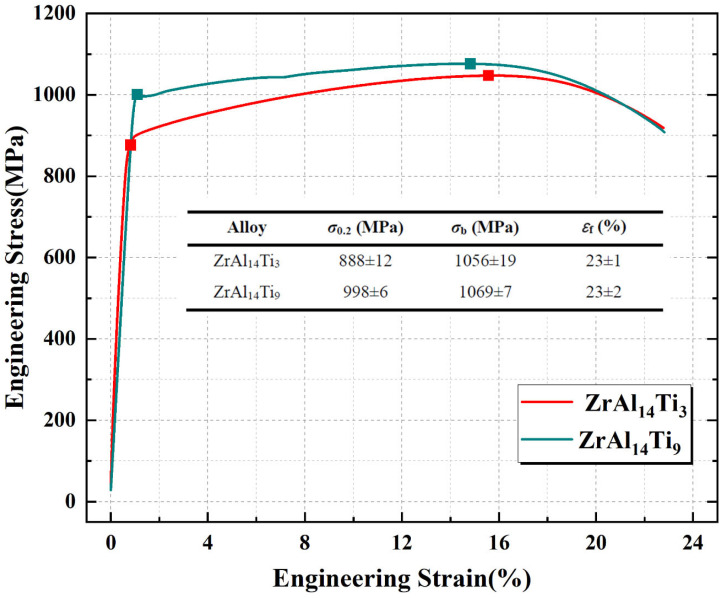
Engineering stress–strain curves of hot-rolled ZrAl_14_Ti_3_ and ZrAl_14_Ti_9_ alloys.

**Figure 13:**
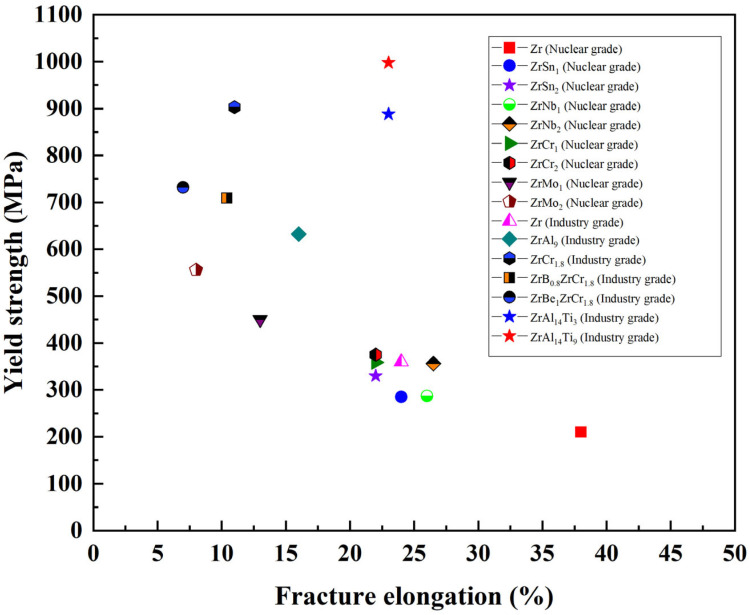
A comparative plot of yield strength versus elongation for present alloys and other representative Zr-based alloys from the literature [7,11,23,24,25,26].

Figure 12 presents the engineering stress–strain curves of the hot-rolled ZrAl_14_Ti_3_ and ZrAl_14_Ti_9_ alloys. The ZrAl_14_Ti_9_ alloy exhibited a yield plateau, indicative of discontinuous yielding, while the ZrAl_14_Ti_3_ alloy showed continuous yielding. The ZrAl_14_Ti_3_ alloy exhibits an offset yield strength (σ_0.2_) of 888 ± 12 MPa, an ultimate tensile strength (σ_b_) of 1056 ± 19 MPa, and a fracture elongation (ε_f_) of 23 ± 1%. Compared with ZrAl_14_Ti_3_, the ZrAl_14_Ti_9_ alloy shows an increase in offset yield strength by 110 MPa (to 998 ± 6 MPa) and in tensile strength by 13 MPa (to 1069 ± 7 MPa), while maintaining the same fracture elongation (23 ± 2%). These properties are highly competitive when compared to other Zr-based alloys reported in the literature [7,11,23,24,25,26], as visually summarized in the comparative plot provided in Figure 13. Microstructural characterization indicates that these variations in strength and ductility are associated with changes in α-Zr solid-solution composition, dislocation density, grain size, texture, and phase fraction. The EBSD results reveal that, relative to ZrAl_14_Ti_3_, the ZrAl_14_Ti_9_ alloy exhibits a higher degree of recrystallization in both α-Zr and Zr_3_Al phases, leading to lower geometrically necessary dislocation densities (Zr_3_Al: 5.28 × 10^13^ m^−2^ vs. 8.45 × 10^13^ m^−2^; α-Zr: 3.44 × 10^14^ m^−2^ vs. 3.79 × 10^14^ m^−2^). This dislocation reduction tends to decrease offset yield strength but enhances ductility. The Zr_3_Al phase fractions in ZrAl_14_Ti_3_ and ZrAl_14_Ti_9_ are 34.0% and 28.9%, respectively; given that the Zr_3_Al phase contains ~25 at.% Al and the total alloy Al content is 14 at.%, the nominal Al contents in the α-Zr matrix are calculated as 8.3 at.% and 9.5 at.%, respectively. The slight difference in Al content suggests that the pronounced shift in α-Zr diffraction peaks toward higher angles in XRD primarily results from increased Ti solubility, confirming the solid-solution strengthening effect of Ti. Another strengthening factor in ZrAl_14_Ti_9_ is grain refinement induced by enhanced recrystallization: the average grain sizes of α-Zr and Zr_3_Al phases decrease from 3.1 µm and 2.1 µm in ZrAl_14_Ti_3_ to 2.6 µm and 1.5 µm, respectively. Using Hall–Petch slopes of 280 MPa·µm^1/2^ [27] for α-Zr and 760 MPa·µm^1/2^ [28] for Zr_3_Al, the grain size changes contribute 19.2 MPa and 135.9 MPa, respectively, to the offset yield strength; considering the phase fractions, grain refinement contributes a net 52.9 MPa to the offset yield strength of ZrAl_14_Ti_9_. Furthermore, the α-Zr phase fraction increases from 66.0% to 71.1% in ZrAl_14_Ti_9_, providing additional strengthening since α-Zr is harder than Zr_3_Al. The lower average Schmid factor for basal slip in the α-Zr of ZrAl_14_Ti_9_ also implies increased orientation hardening. Overall, the offset yield strength improvement in ZrAl_14_Ti_9_ arises from solid-solution strengthening, grain refinement, an increased fraction of the harder α-Zr phase, and orientation hardening. Regarding ductility, although solid-solution strengthening and reduced Zr_3_Al content tend to lower fracture elongation, these effects are offset by grain refinement and the ductility improvement associated with reduced dislocation density.

## 4. Conclusions

This study systematically investigated the microstructure and mechanical properties of hot-rolled ZrAl_14_Ti_3_ and ZrAl_14_Ti_9_ (at.%) alloys. Key conclusions are summarized as follows:

(1) The addition of Ti reduces the dissolution temperature of Zr_3_Al in the α-Zr matrix, enhancing Al solubility in α-Zr and decreasing the equilibrium Zr_3_Al phase fraction.
(2) The addition of Ti promotes the recrystallization of the Zr_3_Al phase and the globularization of the α-Zr phase, refines the grains, and eliminates the basal texture in the α-Zr phase.
(3) The ZrAl_14_Ti_9_ alloy exhibits an offset yield strength of 998 ± 6 MPa, tensile strength of 1069 ± 7 MPa, and fracture elongation of 23% ± 2. Compared with the ZrAl_14_Ti_3_ alloy, the offset yield strength is increased by 110 MPa, the tensile strength is increased by 13 MPa, while the fracture elongation remains unchanged. The main strengthening mechanisms include solid-solution strengthening, grain refinement, an increased fraction of the harder α-Zr phase, and orientation hardening.

## Data Availability

The original contributions presented in this study are included in the article. Further inquiries can be directed to the corresponding authors.
